# Vitamin D deficiency is associated with mortality in the medical intensive care unit

**DOI:** 10.1186/cc10585

**Published:** 2011-12-10

**Authors:** Sindhaghatta Venkatram, Sridhar Chilimuri, Muhammad Adrish, Abayomi Salako, Madanmohan Patel, Gilda Diaz-Fuentes

**Affiliations:** 1 Albert Einstein College of Medicine, Division of Pulmonary and Critical Care Medicine, Bronx Lebanon Hospital Center, 1650 Grand Concourse, Bronx, NY, 10457, USA; 2 Albert Einstein College of Medicine, Department of Medicine, Bronx Lebanon Hospital Center, 1650 Grand Concourse, Bronx, NY, 10457, USA

## Abstract

**Introduction:**

The incidence of vitamin D deficiency in critically ill patients has been reported to range from as low as 17% to as high as 79%. Data regarding the relationship between 25-hydroxyvitamin D levels and outcomes in the medical intensive care unit are sparse. The goal of the study was to evaluate the prevalence of 25-hydroxyvitamin D deficiency in the medical intensive care unit and its relationship with outcomes.

**Method:**

This was a retrospective study in a medical intensive care unit (MICU) at an inner city community hospital. The study period was between October 2009 and February 2010.

**Results:**

Of the 932 patients admitted during the study period, 25-hydroxyvitamin D vitamin D (25(OH)D) levels were available in 523 (53%); 86 of them were excluded from the study due to readmission to the intensive care unit. Deficiency was defined as 0 to 19.9 ng/dL 25(OH)D levels, insufficiency as 20 to 29.9 ng/dL, and normal levels as ≥30 ng/dL. Of the 437 patients studied, 25(OH)D deficiency was identified in 340 (77.8%), insufficiency in 74 (16.9%), and normal levels in 23 (5.3%) patients. Patients with 25(OH)D deficiency/insufficiency were younger (*P *= 0.015), were male (P = 0.001), and had kidney disease (*P *= 0.017) and lower total serum calcium levels (*P *= 0.003). Hospital mortality was higher in patients with 25(OH)D deficiency (*P *= 0.01). No differences in ventilator days or length of stay in the MICU were evident among the three groups. Analysis by multiple logistic regression demonstrated that acute physiology and chronic health evaluation (APACHE) IV score ((odds ratio (OR) 1.036; 95% confidence interval (CI) 1.024-1.048, *P *< 0.0001), ventilator requirement (OR 7.7; 95% CI 4.3-13.98, P < 0.0001), 25(OH) D levels(OR 0.942; 95% CI 0.942-0.904, *P *< 0.0005) and 25(OH) D deficiency (OR 8.7; 95% CI 1.03-72.8, *P *< 0.0469) showed statistical significance. There was no association between 25(OH)]D insufficiency and hospital mortality. The mean 25(OH)D level of survivors (27.9 ± 9.7 ng/dL) was higher than for non-survivors (9.7 ± 4.7 ng/dL; *P *< 0.0001).

**Conclusions:**

The study results demonstrate an association between 25(OH)D deficiency and hospital mortality in MICU patients. A randomized prospective study to evaluate the effect of vitamin D replacement therapy on mortality is warranted.

## Introduction

Vitamin D is a fat-soluble vitamin that regulates calcium metabolism. Adequate levels of 25(OH)D, the storage form of vitamin D, are dependent on cutaneous synthesis stimulated by ultraviolet radiation and/or adequate dietary intake of fortified foods and nutritional supplements. A deficiency in 25(OH)D is estimated to exist in 50% to 60% of the older population in North America and worldwide [1]. Recent evidence suggests that the role of vitamin D is broader than the regulation of calcium metabolism. Vitamin D has been shown to have anti-inflammatory and anti-proliferative properties, and its deficiency has been linked to all-cause mortality and cardiovascular disease and cancer [2-5]. The incidence of 25(OH)D deficiency in critically ill patients has been reported to range from as low as 17% to 79% [6-8]. Data regarding the relationship between 25(OH)D levels and outcome in the medical intensive care unit (MICU) are sparse with new reports suggesting the relationship of deficiency in 25(OH)D and an increase in mortality in the critically ill [6-9].

Most experts agree that levels less than 20 ng/dL 25(OH)D are considered deficient and levels between 20 to 30 ng/dL are insufficient [10-12].

The goal of the study was to evaluate the prevalence of 25(OH)D deficiency in an inner-city MICU. The primary outcome was hospital mortality, and secondary outcomes included duration of mechanical ventilation and MICU length of stay. A subgroup analysis for primary and secondary outcomes was performed for patients admitted from skilled nursing facilities.

## Materials and methods

### Study design and setting

This was a retrospective study of all patients admitted to the MICU between October 2009 and February 2010 at a 26-bed closed unit. Our MICU is a university-affiliated inner-city hospital staffed daily by two pulmonary and critical care-trained attending physicians, a pulmonary fellow, and internal medicine residents.

### Methods

All patients admitted to the MICU who had levels of 25(OH)D available were included in the study. Patients readmitted to MICU during the same period of hospitalization were excluded, those patients either already had 25(OH)D levels available from the first MICU admission and repeated levels were not performed within 24 hours of MICU readmission or did not any level available. The data were collected as part of a performance improvement project looking to the prevalence of 25(OH)D deficiency in our MICU. 25(OH)D levels were collected randomly during the first 24 hours of admission to the intensive care unit. There were no strict criteria to obtain 25(OH)D levels. The physicians received education regarding the high prevalence of hypovitaminosis in our community and were encouraged to evaluate for hypovitaminosis when patients were admitted to the hospital. Baseline demographics (age, gender, and race), history of end stage renal disease (ESRD) or chronic kidney disease (CKD), as well as acute physiology and chronic health evaluation (APACHE) IV were collected. The APACHE derived risk of death during hospitalization was determined from the worst values obtained within 24 hours of MICU admission. Clinical and laboratory variables obtained during the first 24 hours of hospital admission included serum levels of total calcium, phosphate, creatinine, glucose, albumin and 25(OH)D. Utilization and duration of invasive mechanical ventilation, ICU length of stay (LOS), and hospital mortality were analyzed. Length of stay in the MICU was defined as the time from ICU admission to time of transfer out of the MICU. This study was approved by the hospital institutional research review board, and the need for informed consent was waived.

Serum 25(OH)D concentrations were assayed by liquid chromatography-tandem mass spectrometry at Quest Diagnostics, New York, NY. The analytical sensitivity is 4 ng/mL for 25OHD_2 _and 25OHD_3 _with a reportable range of 4 to 512 ng/mL for 25OHD_2 _and 25OHD_3_

### Definition of Vitamin D deficiency

There is no firm consensus regarding optimal levels of 25(OH)D. According to the workshop consensus conference for vitamin D nutritional guidelines and a study that investigated the potential beneficial effects of vitamin D for multiple health outcomes, the minimum desirable serum level of 25(OH)D is suggested to be 20 to 30 ng/dL [1,10,12]. Studies examining 25(OH)D deficiency in intensive care units have no agreement on the cutoff levels for critically ill patients, with deficiency being defined as 25(OH)D levels of less than 15 ng/dL to less than 29 ng/dL [8,9,13]. In our study, we used a 25(OH)D cutoff level of less than 19.9 ng/dL to define 25(OH)D deficiency. 25(OH)D insufficiency was defined as 20 to 29.9 ng/dL [11,14,15].

### Definition of renal failure

There is no consensus on the amount of dysfunction that defines acute kidney injury, with more than 30 definitions in use today [16]. Acute renal failure was defined as a serum creatinine × >1.5 and acute or chronic renal failure as a worsening of renal function in a patient with chronic kidney disease (serum creatinine × 3). End-stage renal disease (ESRD) was defined as any patient with chronic kidney disease on long term hemodialysis [17].

### Statistical analysis

Data analysis was conducted using the SPSS v15.0. Discrete variables are expressed as counts (percentage) and continuous variables as means ± standard deviations (SD). For the demographic and clinical characteristics of the patients, differences between groups were assessed using the chi-squared test and Fisher's exact test for categorical variables and the Student's t-test or Mann-Whitney U test for continuous variables. A one-way analysis of variance (ANOVA) was performed to explore the impact of admitting diagnosis which were classified in nine different categories: (a) Cardiac, (b) Gastrointestinal, (c) Metabolic, (d) Neurologic, (e) Obstructive Airway Disease, (f) others, (g) Pulmonary, (h) Renal, and (i) Sepsis, on the continuous dependent variable outcome of log-Vitamin D levels. A multiple logistic regression model was performed for the whole population, with mortality as the dependent variable, and age, gender, APACHE IV score, ventilator requirement, acute/acute on chronic kidney disease, ESRD, serum levels of total calcium, phosphate, creatinine, and 25(OH)D deficiency and insufficiency as independent variables. Additionally, logistic regression with a dependent variable of mortality was performed with a total of 10 independent continuous variables; (a) APACHE, (b) age, (c) 25(OH)D levels, (d) ventilator days, (e) ICU length of stay, (f) total calcium, (g) phosphate, (h) serum creatinine, (i) serum albumin, and (j) serum glucose. A *P *value less than 0.05 was considered statistically significant. To evaluate the prognostic utility of 25(OH)D levels, a receiver-operating characteristic (ROC) curve was constructed.

## Results

**Figure 1:**
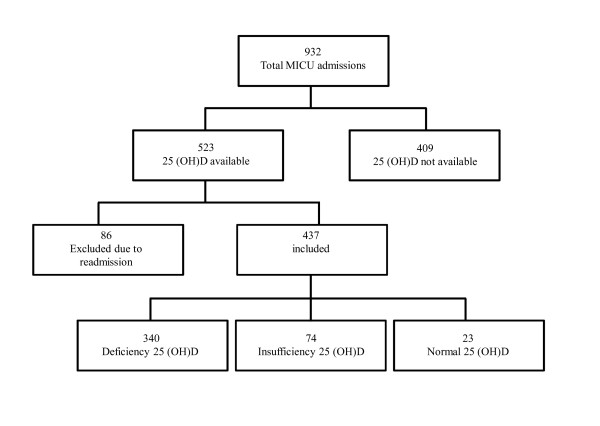
**Flowchart of patients in the cohort**.

A total of 932 patients were admitted to the MICU during the 4-month study period. 25(OH)D levels were available for 523 (56%) patients; of these, 86 patients were excluded due to readmission to the MICU during the same hospitalization. Of the 437 patients studied, 25(OH)D deficiency was identified in 340 (77.8%) patients, insufficiency in 74 (16.9%) patients, and normal levels in 23 (5.3%) patients (Figure 1). Characteristics of the patients were stratified according to 25 (OH)D levels on admission (Table 1). Patients with 25(OH)D deficiency/insufficiency were more likely to be younger (*P *= 0.015), to be male (*P *= 0.001), to have acute/acute on CKD (*P *= 0.017), and to have lower total serum calcium levels (*P *= 0.003). A comparison among the three 25(OH)D groups by admission diagnosis is shown in Table 2. There were no differences in the mean log-25(OH)D levels among the nine categories for admitting diagnosis. (*P *= .099). Comparison of 25-hydroxyvitamin D levels among different admission diagnosis groups did not show statistical significance (Table 3).

**Table 1:** Clinical characteristics of patients with available 25-hydroxyvitamin D levels.

Variable	25(OH)D Deficiency ≤19.9 ng/dL N = 340	25(OH)D Insufficiency 20-29.9 ng/dL N = 74	25(OH)D Normal ≥30 ng/dL N = 23	*P*
Age (years)	55.6 ± 16.5	58.6 ± 19.2	65.4 ± 16.6	0.015
Gender - Male (%)	176 (51.8%)	28 (37.9%)	4 (17.4%)	0.001
APACHE IV	68.3 ± 28.1	60.9 ± 25.9	67.2 ± 20.9	0.11
Ventilator requirement	123 (36.2%)	23 (31%)	7 (30.4%)	0.63
Acute/acute on chronic renal failure	92 (27%)	11 (14.9%)	2 (8.7%)	0.017
ESRD on hemodialysis	21 (7.1%)	6 (8.1%)	2 (8.7%)	0.92
Total calcium serum (mg/dL)	8.6 ± 1.1	9.0 ± 1.1	9.4 ± 1.3	0.0003
Phosphate serum (mg/dL)	3.7 ± 1.7	4.1 ± 5.7	3.3 ± 1.2	0.34

APACHE IV, acute physiology and chronic health evaluation; ESRD, end-stage renal disease; N, Number; 25(OH)D, 25-hydroxyvitamin D

**Table 2:** Comparison of admission diagnosis groups based on deficient, insufficient, and normal 25-hydroxyvitamin D levels.

Variable	25(OH)D Deficiency ≤ 0-19.9 ng/dL N (%) = 340	25(OH)D Insufficiency 20-29.9 ng/dL N (%) = 74	25(OH)D Normal ≥30 ng/dL N (%) = 23	Total N (%)
Cardiac	14(4%)	5(6.7%)	2(8.6%)	21(4.8%)
Gastrointestinal	39(11.4%)	5(6.7%)	3(13%)	47(10.7%)
Metabolic	39(11.4%)	8(10.8%)	3(13%)	50(11.4%)
Neurological	46(13.5%)	6(8.1%)	3(13%)	55(12.5%)
Obstructive airway disease	42(12.3%)	17(22.9%)	1(4.3%)	60(13.7%)
Pulmonary	64(18.8%)	13(17.5%)	3(13%)	80(18.3%)
Others	34(10%)	10(13.5%)	4(17.3%)	48(10.9%)
Renal	19(5.5%)	3(4%)	0(0%)	22(5%)
Sepsis/Septic shock	43(12.6%)	7(9.4%)	4(17.3%)	54(12.3%)

**Table 3:** Comparison of 25-hydroxyvitamin D levels between admission diagnosis groups*

Admission Diagnosis groups	Mean 25-hydroxyvitamin D (ng/dL) ± SD (range)
Cardiac disorders. N (%)	15.1 ± 11.1 (4-43)
Gastrointestinal disorders. N (%)	12.5 ± 10.1 (4-50)
Metabolic disorders. N (%)	13.9 ± 8.9 (4-40)
Neurological disorders. N (%)	12.0 ± 9.5 (4-51)
Obstructive airway disease. N (%)	15.7 ± 10.6 (4-74)
Pulmonary disorders. N (%)	13.1 ± 8.4 (4-42)
Others. N (%)	16.6 ± 11.7 (4-73)
Renal disorders. N (%)	12.2 ± 6.5 (4-26)
Sepsis/Septic shock. N (%)	13.3 ± 9.2 (4-42)

* No statistical significance was reached between the groups

Comparisons of primary and secondary outcomes are shown in Table 4.

**Table 4:** Comparison of outcomes based on deficient, insufficient, and normal 25-hydroxyvitamin D levels.

Variable	25(OH)D Deficiency ≤0-19.9 ng/dL N = 340	25(OH)D Insufficiency 20-29.9 ng/dL N = 74	25(OH)D Normal ≥30 ng/dL N = 23	*P*
Actual Hospital Mortality	82 (24.1%)	9 (12.2%)	1 (4.4%)	0.01
Predicted ICU mortality [APACHE IV]	8.6%	7%	8%	
Days on a ventilator	6.9 ± 6.0	5.9 ± 6.0	6.4 ± 5.1	0.77
ICU length of stay [days]	4.3 ± 4.5	3.7 ± 3.9	4.2 ± 3.7	0.54

APACHE IV, acute physiology and chronic health evaluation; N, number; 25(OH)D, 25-hydroxyvitamin D.

### Primary Outcome

Hospital mortality was higher in patients with 25(OH)D deficiency (*P *= 0.01). Comparisons of observed versus APACHE IV-predicted mortality revealed that the observed mortality was higher than the predicted mortality among patients with 25(OH)D deficiency (24.1% versus 8.6%) and 25(OH)D insufficiency (12.2% versus 7%). However, in patients with normal levels of 25(OH)D, the observed mortality was lower than predicted (4.4% versus 8%).

Early (≤2 days) versus late mortality is shown in Table 5. Classification percentages were similar between the mortality and 25(OH)D groups, but a higher percentage of cases with insufficient 25(OH)D experienced early mortality (13.8%) than late mortality (7.9%). A higher percentage of cases with deficient 25(OH)D levels were classified as late mortality (69.5%) versus early mortality (30.5%) (P < 0.0005)

**Table 5:** Contingency table of cross tabulations between classifications of Vitamin D levels and stages of mortality (N = 92).

	25(OH)D Deficiency ≤ 0-19.9 ng/dL N = 82	25(OH)D Insufficiency 20-29.9 ng/dL N = 9	25(OH)D Normal ≥30 ng/dL N = 1	Total
**Early Mortality (frequency)**	25	4	0	29
				
% within mortality category	86.2	13.8	0.0	100.0
% within Vitamin D category	30.5	44.4	0.0	31.5
% of total	27.2	4.3	0.0	31.5
				
**Late Mortality (frequency)**	57	5	1	63
				
% within mortality category	90.5	7.9	1.6	100.0
% within Vitamin D category	69.5	55.6	100.0	68.5
% of total	62.0	5.4	1.1	68.5

N, number

Unadjusted and adjusted odds ratios (ORs) for mortality are shown in Table 6. The following variables demonstrated statistical significance after adjustment by multiple logistic regression analysis: APACHE IV score (OR 1.036; 95% confidence interval (CI) 1.024-1.048, *P *< 0.0001), ventilator requirement (OR 7.7; 95% CI 4.3-13.98, *P *< 0.0001), and 25(OH)D deficiency (OR 8.7; 95% CI 1.03-72.8, *P *< 0.0469). No association between 25(OH)D insufficiency and hospital mortality (OR 4.3; 95% CI 0.4-40.9, *P *= 0.2081) was evident.

**Table 6:** Logistic regression analysis for mortality risk using categorical variables.

Variable	Unadjusted OR for Death	*P*	Adjusted OR for Death	95% CI	*P*
Ventilator requirement	11.7	<0.0001	7.7	4.3-13.98	<0.0001
25(OH)D deficiency	6.99	0.0289	8.7	1.03-72.8	0.0469
25(OH)D insufficiency	3.05	0.29	4.3	0.4-40.9	0.2081

APACHE IV, acute physiology and chronic health evaluation; CI, confidence interval; 25(OH)D, 25-hydroxyvitamin D; OR, odds ratio.

Logistic regression with a dependent variable of mortality and continuous variables was performed for greater retention of information during analysis (Table 7). Wald statistics indicated that four variables contributed significantly to the model: APACHE (χ2 = 29.01, OR 1.037; 95% CI 1.023-1.050, *P *< .0005), 25(OH) D levels (χ2 = 8.083, OR 0.94; 95% CI 0.904-0.982, *P *= 0.004), admission albumin levels (χ2 = 13.27, OR 0.457; 95% CI 0.0.300-1.091, *P *< 0.0005), and ventilator days (χ2 = 9.2, OR 1.15; 95% CI 1.054-1.272, *P *= 0.002).

**Table 7:** Logistic regression analysis for mortality risk using continuous variables.

Variable	Wald *χ^2^*	*P*-value	OR	95% CI
APACHE	29.013	<.0005	1.037	1.023-1.050
25(OH)D	8.083	.004	0.942	0.904-0.982
Ventilator days	9.285	.002	1.158	1.054-1.272
Albumin	13.276	<.0005	0.457	0.300-1.091
Age	0.432	.511	1.007	0.987-1.027
ICU LOS	0.015	.903	1.007	0.906-1.118
Total calcium	0.082	.775	0.963	0.745-1.245
Phosphate	0.047	.828	1.012	0.910-1.125
Creatinine	0.580	.446	0.946	0.821-1.125
Glucose	1.778	.182	0.999	0.997-0.696

APACHE IV, acute physiology and chronic health evaluation; CI, confidence interval; 25(OH)D, 25-hydroxyvitamin D; LOS, Length of stay; OR, odds ratio.

**Figure 2:**
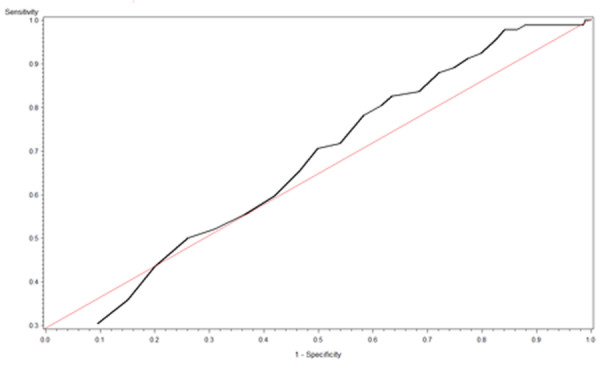
**Vitamin D receiver operating curve**. Vitamin D receiver operating characteristic curve revealing an area under the curve of 0.66.

**Figure 3:**
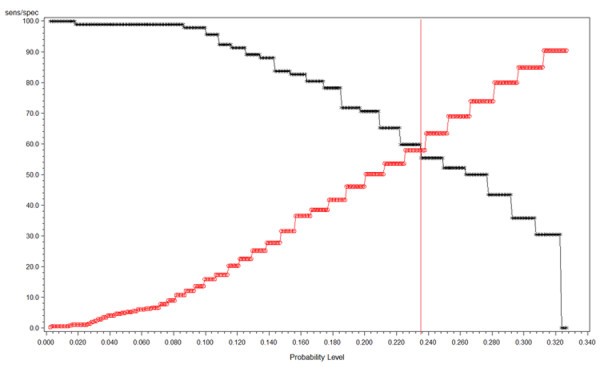
**Vitamin D intersection curve**. Vitamin D intersection curve revealing sensitivity at 59.8% and specificity at 58% for a vitamin D level of 10 ng/dL.

The mean 25(OH)D level for survivors (27.9 ± 9.7 ng/dL) was higher than for non-survivors (9.7 ± 4.7 ng/dL; *P *< 0.0001). The ROC curve for 25(OH)D levels is shown in Figure 2, and the 25(OH)D intersection curve is shown in Figure 3. The area under the curve (AUC) was 0.66, and the cut-off value that maximizes sensitivity at 59.8% and specificity at 58% is a 25(OH)D level of 10 ng/dL. Sensitivity and specificity crossed at a probability level of 0.235 for the aforementioned 25(OH)D level. With a prevalence rate of 77.8%, the positive predictive value (PPV) for mortality with a 25(OH)D level less than 10 ng/dL was 83.64% and the negative predictive value (NPV) was 29.52%..

### Secondary Outcomes

Factors that did not significantly differ between stratified groups were latitude, ventilator days and MICU length of stay. The mean latitude for our study group was 40° north (SD 0.5° north). There was no difference in ventilator days or MICU LOS for the different admitting diagnosis groups.

**Figure 4:**
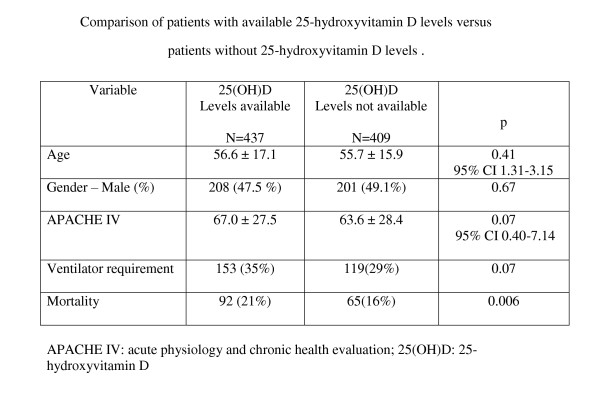
**Comparison of patients with available 25-hydroxyvitamin D levels versus patients without 25-hydroxyvitamin D levels**.

Comparison of patients with available 25(OH)D levels versus patients without 25(OH)D revealed no difference for age, gender, APACHE IV score, ventilator requirement or mortality (Figure 4).

## Discussion

Our results demonstrate that 56% of patients admitted to our MICU have a 25(OH)D deficiency. This incidence is higher than other large studies showing a prevalence between 20% to 40% [6,8,9]. Inner-city hospitals are unique in the sense that they care for an underserved population with a higher rate of unemployment, lower income, higher use of toxic substances and, in general, less than optimum medical care; all of which lead to an increased incidence of uncontrolled diseases and a higher risk for hypovitaminosis.

We found an association between 25(OH)D deficiency and hospital mortality in our MICU population. 25(OH)D levels were significantly higher in survivors than in non-survivors. In our study, total serum hypocalcemia was not associated with an increased hospital mortality, although levels of ionized calcium, 25(OH)D3 and PTH were not studied. Our data are in line with another study showing a correlation between serum levels of albumin and mortality [5]. Several explanations are possible for the association between 25(OH)D deficiency and hospital mortality. The vitamin D receptor is expressed in nearly all cells in the body, and the activating enzyme 1-alpha-hydroxylase is expressed in many tissue types. Laboratory, cell culture, and animal studies suggest that vitamin D may lower cancer risk by inhibiting cell proliferation, angiogenesis, metastasis, and inflammation, as well as inducing apoptosis and cellular differentiation. Several of these mechanisms are relevant to atherosclerosis and cardiovascular disease, as well as sepsis, respiratory failure, and other diseases commonly seen in the critically ill [12,18-20]. Deficiency of 25(OH)D has been implicated as a cause of increased cardiovascular events and death [21-24]. The increased mortality in the critically ill with vitamin D deficiency might be due to changes in glucose and calcium metabolism, and/or immune and endothelial cell dysfunction due to the deficiency [25-29].

Endothelial cell dysfunction has been proposed as a potential cause of multiple organ dysfunction syndrome [30-32]. It is possible that 25(OH)D deficiency amplifies the metabolic derangements and impaired immune regulation seen in critically ill states, which may lead to worse outcomes than would be experienced with normal vitamin D levels. Furthermore, 25(OH)D deficiency has been implicated in sepsis, stroke, inflammatory bowel disease, autoimmune conditions and asthma [33-39].

Contrary to the study by McKinney *et al*., we did not find a correlation between 25(OH)D deficiency and an increased length of stay among patients admitted to the MICU; it is important to note that in their study the LOS was dichotomized to a LOS less and more than three days, respectively [8].

Risk factors for low vitamin D levels include older age, living in northern latitudes, sun avoidance, dark skin pigmentation, obesity, low dietary intake of vitamin D, and various medical conditions, especially malabsorption syndromes. These factors are especially important for older patients in nursing home facilities [40].

Causes of low 25(OH)D levels in patients admitted to ICUs are multifactorial. In addition to the well-known etiologies, it is important to consider other factors such as interaction with medications, abnormal gastrointestinal function and the effect of fluid resuscitation [41].

Contrary to our expectations and reports in the literature, our study showed that patients with either 25(OH)D deficiency or insufficiency were generally younger than patients with normal 25(OH)D levels and they were predominantly of male gender. The association between 25(OH)D levels and hospital mortality in men and in younger patients is unclear. Most published studies show a higher prevalence of vitamin D deficiency in women and the elderly [9,42,43]. The large multicenter study done by Braun *et al*. confirmed our association between low 25(OH)D levels and younger age, but not with male gender [9]. These findings could be just a reflection of the general vitamin deficiency in our population.

In our cohort, 93% of patients with ESRD and 98% of patients with acute and acute on CKD had 25(OH)D deficiency/insufficiency, and these findings are consistent with other published findings [14,44-49]. Chronic kidney disease is characterized by decreased renal phosphate excretion, with resultant increases in serum phosphate levels; furthermore, there is decreased conversion of vitamin D to its active form, 1,25-dihydroxyvitamin D3 (1,25(OH)D3), resulting in decreased levels of circulating 1,25(OH)D3 and serum calcium and decreased intestinal calcium absorption. The hyperphosphatemia, hypocalcemia, and decreased levels of active vitamin D result in increased synthesis and secretion of parathyroid hormone. Some studies found no interaction between low levels of 25(OH)D and PTH concentrations or calcium levels. This could suggest that the association of 25(OH)D status and mortality is not significantly modified by PTH or calcium levels [6,47,50]. Vitamin D deficiency has been associated with cardiovascular mortality and all-cause mortality in patients with CKD [44-47,50-52]. There is no conclusive data regarding vitamin D supplementation and decrease in mortality or other outcomes in critically ill patients. A meta-analysis of randomized controlled trials suggested that supplementation of 400 to 830 IU of vitamin D decreased mortality in the general population during the trial periods [21]. In a subsequent study, there was no association between vitamin D classes and mortality [6]. Levels of 25(OH)D ≥150 ng/dL are potentially harmful and are associated with elevated risk of hypercalcemia, vascular soft tissue calcification, and hyperphosphatemia [53]. Vitamin D intoxication can potentially be life-threatening but the majority of officially recorded cases could be related to prolonged intakes of >40,000 IU per day [54]. One small study looking at the short-term metabolic effect of high dose oral vitamin D3 replacement in the intensive care unit did not reveal any complications [55]. A recent Cochrane review of fifty randomized trials with 94,148 participants showed that vitamin D in the form of vitamin D_3 _seems to decrease mortality in predominantly elderly women [56].

Our work has several potential limitations. First, this was a retrospective single center study and we did not sample 25(OH)D levels sequentially. The 25(OH)D levels obtained on admission are probably a reflection of pre-admission deficiency. Vitamin D levels were not available for all patients in this cohort; however, analysis of the groups with and without available vitamin D levels reflected no gross bias. Second, the study was completed in the fall and winter months, which have been traditionally associated with lower levels of vitamin D, and may have overestimated the deficiency of vitamin D in our population. Third, our study was conducted in a MICU and cannot be generalized to cardiac, surgical, or cardiothoracic units. Fourth, our study did not intend to evaluate the association of low 25(OH)D levels and inflammatory markers or incidence of infectious diseases, neither did we attempt to see the effect of 25(OH)D replacement on mortality. Finally, PTH and 1,25D3 levels were not available and we cannot exclude the confounding effects of these variables.

## Conclusions

In conclusion, we report on a large cohort of patients with 25(OH)D deficiency and insufficiency in a MICU setting. Our study shows a clear association between 25(OH)D levels and hospital mortality in critically ill patients. 25(OH)D levels of 10 ng/dL predicted hospital mortality in 83.6% of this cohort. The observed hospital mortality for 25(OH)D deficient patients was higher than the predicted mortality based on admission APACHE IV score.

The finding that 25(OH)D deficiency, especially at levels less than 10 ng/dL, is associated with increased hospital mortality has both clinical and research implications. Clinically, patients admitted to the medical ICU who present with a 25(OH)D deficiency are at greater risk for short-term hospital mortality and may therefore potentially benefit from more intensive surveillance at ICU admission. Future studies are needed to answer some of the most relevant questions such as: Is 25(OH)D deficiency merely another marker for severity of illness? Can hospital mortality, risk of infections, LOS in the intensive care unit be changed or modulated just by evaluating and correcting 25(OH)D deficiency ? What are the optimal doses for replacement and what is the long term outcome in those patients? Future research is warranted to determine whether correction of 25(OH)D deficiency is associated with improved outcomes for ICU patients.

## Key Messages

• 25(OH)D deficiency and insufficiency is a common finding in a medical intensive care unit.

• 25(OH)D deficiency in the intensive care unit is associated with increased risk for hospital mortality.

• There was correlation between 25(OH)D deficiency and late mortality (≥2 days) whereas this effect was not seen in 25(OH)D insufficiency.

• A 25(OH)D level less than 10 ng/dL had a positive predictive value for hospital mortality of 83.6%.

• Measurement of 25(OH)D D levels should be considered as part of the routine initial laboratory tests obtained in the medical intensive care unit.

## Abbreviations

ANOVA: one-way analysis of variance; APACHE: acute physiology and chronic health evaluation; CKD: chronic kidney disease; ESRD: end stage renal disease; ICU: intensive care unit; MICU: medical intensive care unit; 25(OH)D: 25-hydroxyvitamin D; OR: odds ratio; PTH: parathyroid hormone; ROC: receiver-operating characteristic; SD: standard deviation.

## Competing interests

The authors declare that they have no competing interests.

## Authors' contributions

SV conceived the study, and participated in its design and coordination and helped to draft the manuscript, SC was involved in revising the manuscript critically for important intellectual content, MA made substantial contributions in the acquisition, analysis and interpretation of data, AS made substantial contributions in the acquisition and analysis of data, MP made substantial contributions in the acquisition of data and analysis and interpretation of data, GDF participated in the study design and coordination and helped to draft the manuscript. She gave final approval of the version to be published. All authors read and approved the final manuscript.
